# Maternal sepsis in pregnancy and the puerperal periods: a cross-sectional study

**DOI:** 10.3389/fmed.2023.1126807

**Published:** 2023-05-16

**Authors:** Ping Liu, Xiaowei Zhang, Xinxin Wang, Yiheng Liang, Nan Wei, Zhansong Xiao, Ting Li, Ruilian Zhe, Weihua Zhao, Shangrong Fan

**Affiliations:** ^1^Department of Obstetrics and Gynecology, Peking University Shenzhen Hospital, Shenzhen, Guangdong, China; ^2^Shenzhen Key Laboratory of Gynecological Diagnostic Technology Research, Peking University Shenzhen Hospital, Shenzhen, China; ^3^Department of Obstetrics and Gynecology, Suzhou Municipal Hospital, Suzhou, China; ^4^Department of Obstetrics, Shenzhen People’s Hospital, Shenzhen, China; ^5^Department of Obstetrics, Shenzhen Second People’s Hospital, The First Affiliated Hospital of Shenzhen University Health Science Center, Shenzhen, China

**Keywords:** maternal sepsis, infection, maternal and fetal outcome, organ failure, obstetrical critical illness

## Abstract

Maternal sepsis is a life-threatening condition and ranks among the top five causes of maternal death in pregnancy and the postpartum period. Herein, we conducted a retrospective study on sepsis cases to explain the related risk factors by comparing them with bloodstream infection (BSI) and control maternities. In total, 76 sepsis cases were enrolled, and 31 BSI and 57 maternal cases of the same age but with neither sepsis nor BSI were set as controls. Genital tract infection (GTI) and pneumonia were the two most common infection sources in both sepsis (22 cases, 29% and 29 cases, 38%) and BSI cases (18 cases, 58% and 8 cases, 26%). Urinary tract infection (UTI)/pyelonephritis (9 cases, 12%) and digestive infection cases (11 cases, 14%) only existed in the sepsis group. Significantly different infection sources were discovered between the sepsis-death and sepsis-cure groups. A higher proportion of pneumonia and a lower proportion of GTI cases were present in the sepsis-death group (17 cases, 45% pneumonia and 9 cases, 24% GTI) than in the sepsis-cure group (12 cases, 32% pneumonia and 13 cases, 34% GTI). In addition, although gram-negative bacteria were the dominant infectious microorganisms as previously reported, lower proportion of gram-negative bacteria infectious cases in sepsis (30 cases, 50%) and even lower in sepsis-death group (14 cases, 41%) was shown in this study than previous studies. As expected, significantly greater adverse maternal and fetal outcomes, such as higher maternal mortality (26.3% vs. 0% vs. 0%), higher fetal mortality (42.2% vs. 20.8% vs. 0%), earlier gestational age at delivery (26.4 ± 9.5 vs. 32.3 ± 8.1 vs. 37.7 ± 4.0) and lower newborn weight (1,590 ± 1287.8 vs. 2859.2 ± 966.0 vs. 3214.2 ± 506.4), were observed in the sepsis group. This study offered some potential pathogenesis and mortality risk factors for sepsis, which may inspire the treatment of sepsis in the future.

## 1. Introduction

Maternal sepsis, a life-threatening condition defined as organ dysfunction resulting from obstetric infections during pregnancy, childbirth, postabortion, or postpartum period, is a major public health concern and one of the top five causes of maternal death in pregnancy and postpartum periods (1–4). Direct obstetric infections are the third most common cause of maternal mortality globally, representing approximately 10.7% of all maternal deaths, among which the mortality rate of severe sepsis is 7–8%, while it can increase to 20–40% with organ dysfunction and increase to 60% if shock occurs (5, 6). Sepsis in pregnancy and postpartum periods not only causes prolonged hospitalization but also remains the main cause of maternal mortality in both developed countries, such as the United States, and developing countries, such as sub-Saharan Africa (6–9). In the United States, maternal sepsis complicated 0.04% of deliveries. Of all maternal deaths, over 20% were sepsis-related, and it ranks first among all causes of maternal death according to the reported data from 2003 to 2011 (10, 11). Fortunately, the mortality rate of severe sepsis in developed countries is controlled below 5% (4.4–4.6%) following the introduction of antibiotic therapy, improvement in social infrastructure, and systematic use of infection control measures in healthcare (12). In sub-Saharan Africa, which has the highest maternal mortality worldwide, sepsis is responsible for 130,000 maternal deaths annually (13). A prompt and appropriate diagnosis and treatment are needed to improve maternal and fetal morbidity and mortality (2, 14). Therefore, more case studies are needed to uncover the high probability cause factors of sepsis induction and treatment strategies, especially in developing countries. Herein, we conducted a retrospective study on the clinical characteristics of sepsis, causative microorganisms, and the outcomes of women and their fetuses to explain the related risk factors by comparison with bloodstream infection and healthy maternities.

## 2. Materials and methods

### 2.1. Study population

**Figure 1:**
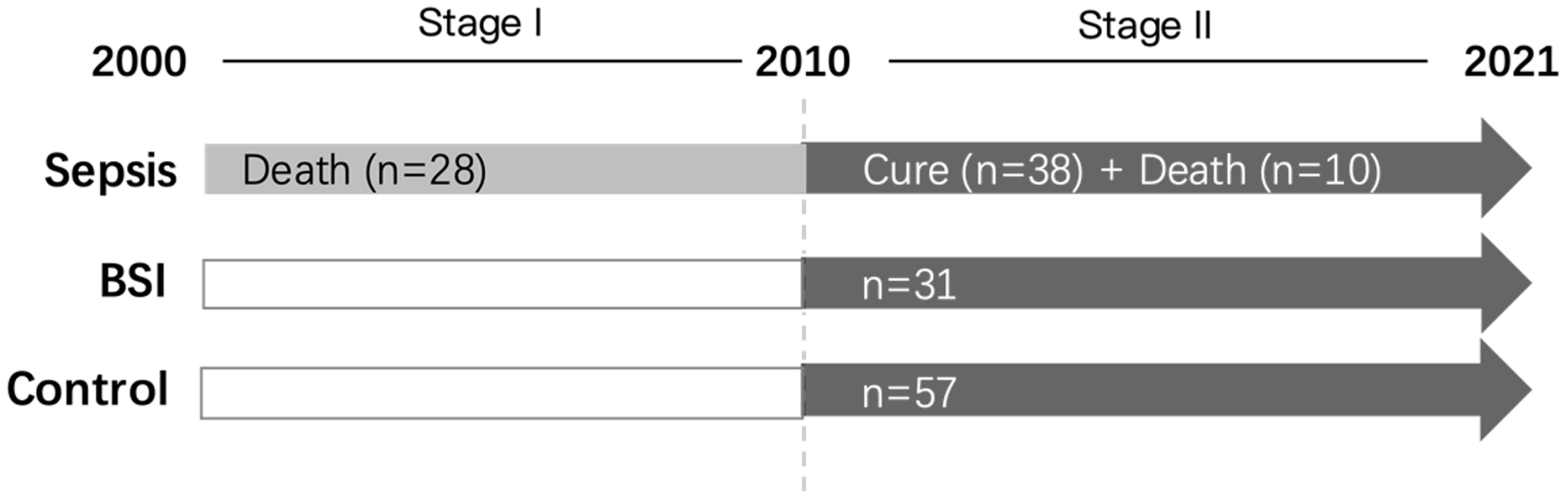
The workflow of case collection in this study. The collection period can be divided into two stages, including stage I (2000–2021) and stage II (2010–2021). During stage I, only maternal sepsis death cases were collected, and 28 sepsis death cases from 12 hospitals in China were enrolled in total. For stage II, 48 symptomatic cases of maternal sepsis were collected, among which 38 cases were cured and 10 died. In addition, 31 patients with bloodstream infections (BSIs) and 57 maternal cases of the same age who were diagnosed with neither sepsis nor BSI were enrolled as the control groups.

In total, we collected symptomatic cases of maternal sepsis from 2000 to 2021. The collection period can be divided into two stages, including stage I from 2000 to 2010 and stage II from 2011 to 2021. During stage I, only maternal sepsis death cases were collected, and 28 sepsis death cases from 12 hospitals in China were enrolled in total. For stage II, 48 symptomatic cases of maternal sepsis during 2010–2021 in Peking University Shenzhen Hospital, Shenzhen University First Hospital, Jinan University Second Hospital, and Suzhou Municipal Hospital were collected, among which 38 cases were cured and 10 died (Figure 1). All of the sepsis cases were defined as follows: (1) maternal infection; and (2) Sequential Organ Failure Assessment (SOFA) score ≥ 2 points except for other causes, which is a clinical tool that evaluates the severity of illness and organ dysfunction in critically ill patients, and is based on six organ systems and ranges from 0 to 24, with higher scores indicating greater dysfunction. Septic shock was indicated as persistent tissue hypoperfusion even with adequate fluid (2, 15, 16). In addition, 31 patients with bloodstream infections (BSI), defined as positive blood cultures but not meeting the case definitions of sepsis in Peking University Shenzhen Hospital, were set as controls. Additionally, 57 maternal cases of the same age but who were neither diagnosed with sepsis nor BSI were enrolled as another control group. The study was approved by the Peking University Shenzhen Hospital Scientific Research and Ethical Committee on 3/3/2020 (reference number: 2020/476). The ethics of other hospitals were waived because of the minimal risk conveyed by the study to the participants as per the hospital’s regulations.

### 2.2. Data collection

Demographic, clinical, microbiological and outcome data were obtained according to the patient’s medical records. Among them, demographic characteristics included age, obstetric history, and onset period. The clinical features included initial infection sites, organ dysfunction, and the occurrence of obstetrical critical illness (OCI). OCI is characterized as any acute illness, complication, or comorbidity experienced by women during pregnancy, childbirth, or within the 42-day after delivery, such as severe postpartum hemorrhage, eclampsia, severe preeclampsia, amniotic fluid embolism, organ failure, etc. The initial infection sites were defined according to the primary symptoms when the patients came to the hospital and classified into five groups, including genital tract infection, pneumonia, urinary tract infection (UTI)/pyelonephritis, digestive tract infection, and others. Organ dysfunction, such as circulatory dysfunction, respiratory dysfunction, renal dysfunction, liver dysfunction, and disseminated intravascular coagulation (DIC), was also recorded. Among them, DIC is defined as a serious condition where abnormal blood clotting and bleeding occur throughout the body, leading to tissue damage and organ failure. The culture results from blood, intrauterine fluid, ascitic fluid, chest water, and bone marrow were identified as microbiological data. In addition, maternal and fetal outcomes, such as gestational age at delivery, mode of delivery, mode of membrane rupture, amniotic fluid turbidity, pregnancy outcomes, newborn weight, and Apgar scores at 1 and 5 min, were obtained.

### 2.3. Statistical analysis

Patient characteristics are described as proportions for categorical variables and mean and standard deviation (SD) for continuous variables. Significant differences between groups were examined by chi-square and Fisher’s exact tests for categorical variables and one-way ANOVA with *post hoc* Bonferroni correction for continuous variables. Data analyses were performed using the statistical software package SPSS 28.0 (SPSS Inc., Chicago, IL, United States).

## 3. Results

### 3.1. Study population

A total of 76 patients with sepsis, 31 patients with BSI and 57 controls were included in the study. Among sepsis cases, 38 were cured and were all collected after 2010, while 38 sepsis cases were dead, among which 28 were collected before 2010 and 10 after 2010. The ages of the sepsis, BSI, and control groups were 29.8 ± 5.9, 31.1 ± 4.3, and 31.5 ± 5.0 years, respectively, and showed no significant difference between groups. Additionally, no significant difference was detected within sepsis subgroups (Table 1). The obstetric history of the participants was also collected without missing data. The number of previous cesarean sections in the sepsis group was significantly higher than that in the BSI and control groups (*p* < 0.001), but no significant difference was shown between the sepsis-cure and sepsis-death groups. Other obstetric histories, including numbers of pregnancies, extrauterine pregnancy, spontaneous delivery, and spontaneous abortion, were not significantly different between groups, either between the sepsis-cure or sepsis-death groups. Among all the cases, only one case experienced an induced abortion, which was presented in the sepsis-cure group (Table 1). For BSI cases, the highest onset rate appeared in the postpartum period or after abortion, which accounted for up to 67.7%. It was followed by late pregnancy and the second trimester, and no BSI cases occurred in the first trimester. Sepsis cases appeared throughout the pregnancy and postpartum periods, so the onset period was recorded with two missing data of sepsis-death cases. Among them, 24 cases occurred in late pregnancy, 22 cases in the second trimester, 5 cases in early pregnancy, and 23 cases in the postpartum period or after abortion. One postpartum onset case experienced sepsis after drug treatment and surgical treatment of ectopic pregnancy. The onset period was significantly different among the sepsis, BSI, and control groups, but none existed between the sepsis-cure and sepsis-death groups (Table 1).

**Table 1:** Demographic characteristics of the patients with sepsis, BSI, and controls, as well as in sepsis subgroups including sepsis-cure and sepsis-death groups.

Characteristics	All groups					
	Sepsis			BSI (*n* = 31)	Control (*n* = 57)	Statistical significance
	Sepsis-death (*n* = 38)	Sepsis-cure (*n* = 38)	Sepsis-total (*n* = 76)			
Age (SD)	29.5 ± 5.9	30.2 ± 6.0	29.8 ± 5.9	31.1 ± 4.3	31.5 ± 5.0	ns
Obstetric history (No.)						
Pregnancy	23 (46.94%)	16 (45.71%)	39 (46.43%)	17 (36.96%)	41 (47.13%)	*, †
Extrauterine pregnancy	1 (2.04%)	1 (2.86%)	2 (2.38%)	0	0	ns
Spontaneous delivery	11 (22.45%)	5 (14.29%)	16 (19.05%)	7 (15.22%)	9 (10.34%)	ns
Cesarean	4 (8.16%)	3 (8.57%)	7 (8.33%)	10 (21.74%)	15 (17.24%)	**, ##, ††
Spontaneous abortion	10 (20.41%)	9 (25.71%)	19 (22.62%)	12 (26.09%)	22 (25.29%)	ns
Induced abortion	0	1 (2.86%)	1 (1.19%)	0	0	ns
Onset period						
1st trimester	4 (11.1%)	1 (2.6%)	5 (6.8%)	0	0	*
2nd trimester	10 (27.8%)	12 (31.6%)	22 (29.7%)	2 (6.5%)	3 (5.3%)	***, ##, †††
3rd trimester	10 (27.8%)	14 (36.8%)	24 (32.4%)	8 (25.8%)	54 (94.7%)	***, †††, δδδ
Postpartum/after abortion	12 (33.3%)	11 (28.9%)	23 (31.1%)	21 (67.7%)	0	***, ###, †††, δδδ

Data of continuous variables are presented as the mean and standard deviation (SD), and the categorical variable data are presented as counts and percentages. Symbol * represent the significance of three groups (sepsis, BSI and control). Symbols #, †, δ represent the significance of sepsis vs. BSI, sepsis vs. control, and BSI vs. control, respectively. One symbol denotes a value of *p* < 0.05. Two symbols denote a value of *p* < 0.01. Three symbols denote *p-*value < 0.001.

### 3.2. Infection sources

**Figure 2:**
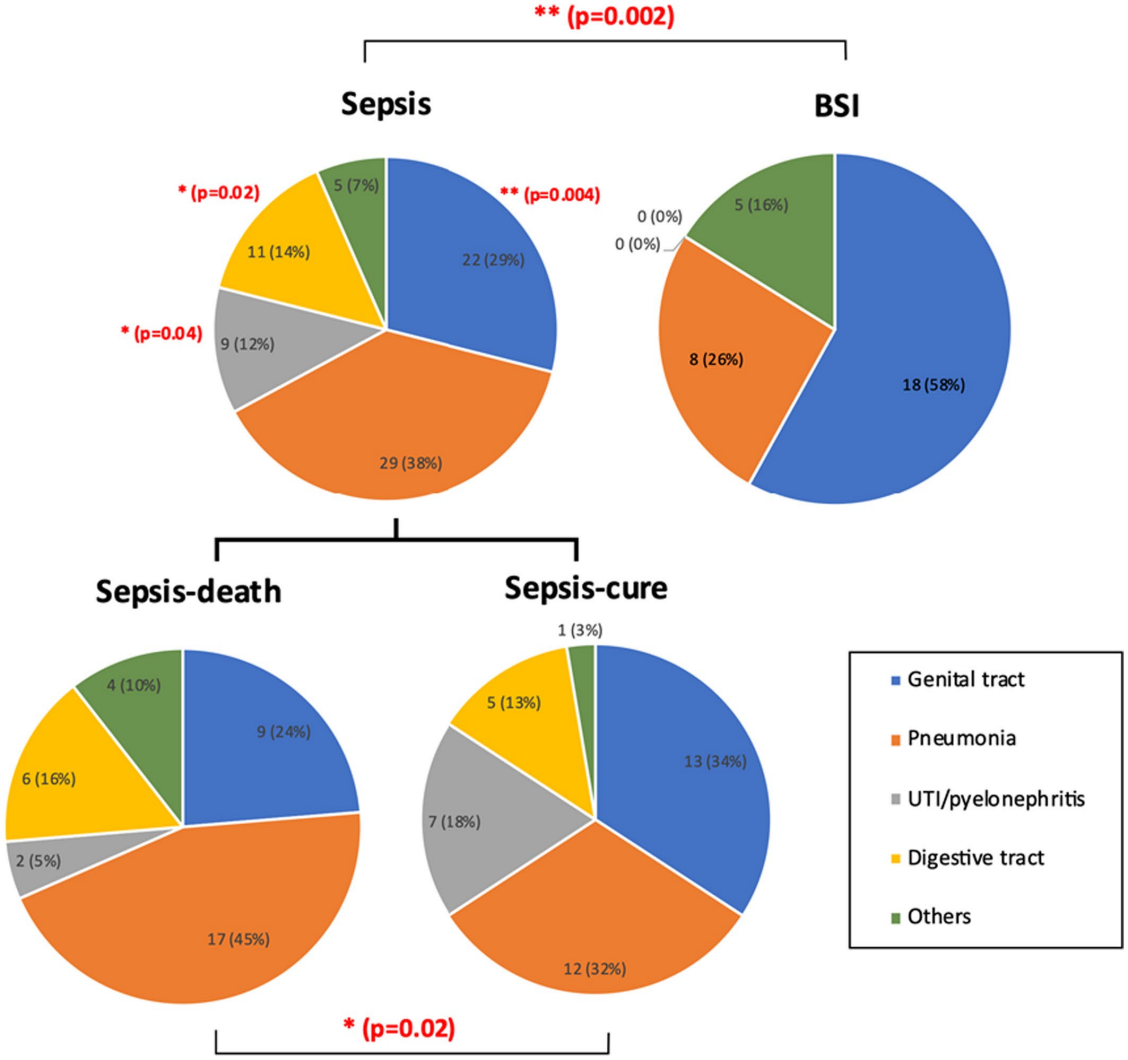
The infection sources were significantly different between the sepsis and BSI groups, as well as between the sepsis-cure and sepsis-death groups. UTI/pyelonephritis and digestive infection showed significantly higher frequency in sepsis patients than in BSI patients, while significantly more genital tract infection cases were shown in BSI patients. Pneumonia may be a risk factor for death in sepsis patients. The case number and ratio are labeled in the figure. *p*-values with significant changes are marked as red text. The types of diseases included in each group are presented in Supplementary Table S1.

The infection sources were recorded according to the initial related symptoms and clinical test results. Significant differences were found both between the sepsis and BSI groups and between the sepsis-cure and sepsis-death groups. In general, genital tract infection and pneumonia were the two most common causes of sepsis and BSI, and the number of BSI cases caused by genital tract infection (58%, 18 cases) was significantly higher than that caused by sepsis (29%, 22 cases). Urinary tract infection (UTI)/pyelonephritis showed a significantly higher frequency in sepsis patients than in BSI patients. In addition, digestive infection cases only existed in the sepsis groups but not in the BSI group (Figure 2). The proportion of pneumonia cases accounted for up to 45% (17 cases) in the sepsis-death group, which was higher than that in the sepsis-cure group (32%, 12 cases). This may indicate that pneumonia may lead to higher mortality of sepsis. However, the proportions of cases with genital tract infection in the sepsis-cure group were higher than those in the sepsis-death group (34% vs. 24%) (Figure 2).

### 3.3. Microbial features

Microorganisms from blood, intrauterine fluid, ascitic fluid, chest water, and bone marrow were cultured and identified. Urine, vaginal discharge, sputum, bronchoalveolar fluid, and other easily contaminated samples were not analyzed in this study. In total, 91 microorganisms were cultured from 64 patients, including 60 strains from 37 sepsis cases and 31 strains from 27 BSI cases, which demonstrated a higher complexity of infection in sepsis patients. All BSI cases had positive blood culture results, and 2 cases were complicated with ascites and intrauterine infection. All 37 sepsis patients also had positive blood culture results, and 8 of them were also positive in other cultures.

Among all 91 cultured microorganisms, 6 were fungi and 85 were bacteria. *Escherichia coli* was the most frequent bacteria, accounting for 30.6% (26 strains) of the total microorganisms that affected 24 patients (37.5%), and two of them were third- and fourth-generation cephalosporin-resistant strains. Enterococcus coli, *Enterococcus faecalis* (8, 8.8%), *Acinetobacter baumannii* (7, 7.7%), *Klebsiella pneumoniae* (6, 6.6%), Staphylococcus spp. (5, 5.5%) and *Listeria monocytogenes* (4, 4.4%) were the other common bacterial pathogens. In addition, Candida was the most common pathogenic fungus, accounting for 83.3% (5 strains) of all cultured fungal strains.

**Figure 3:**
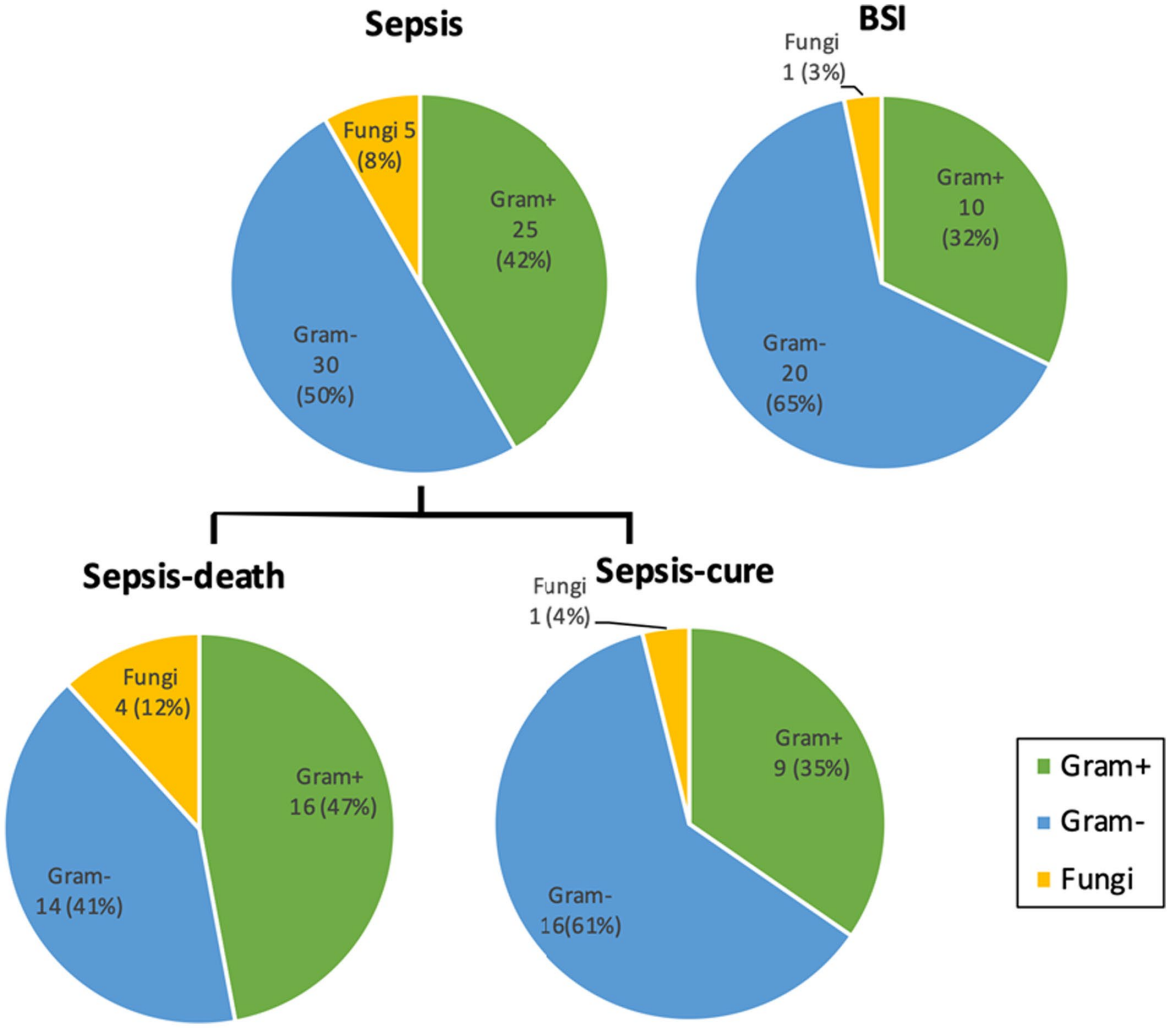
The percentages of gram-positive bacteria and fungi cultured from sepsis patients were both higher than those from the BSI group and higher from the sepsis-death group than from the sepsis-cure group. Microorganism numbers and ratios were labeled. No significant difference was detected between the groups. Detailed information on the cultivated microorganisms is presented in Supplementary Table S2.

For all the cultured microorganisms, gram-negative bacteria were the largest group, accounting for 50 and 65% in the sepsis and BSI groups, respectively. However, the percentage of gram-negative bacteria infectious cases in sepsis group was lower in this study than previously reported, and even lower in the sepsis-death group. Gram-positive bacteria and fungi accounted for 42% (25 strains) and 8% (5 strains) in the sepsis group, respectively, and both were higher than those in the BSI group, but no significant difference was shown. Additionally, the percentages of gram-positive bacteria and fungi were higher in the sepsis-death group than in the sepsis-cure group (47% vs. 35% and 12% vs. 4%), which demonstrated that higher risks of failure treatment may occur in the infection cases of gram-positive bacteria and fungi (Figure 3).

### 3.4. Maternal and fetal outcomes

The maternal and fetal outcomes of cases in the sepsis, BSI, and control groups were compared, as well as the sepsis-death and sepsis-cure cases (Table 2). The gestational weeks of sepsis cases were 26.4 ± 9.5, which was significantly earlier than that of control cases (37.7 ± 4.0) and was also earlier than BSI cases (32.3 ± 8.1) but with no significance. BSI cases had a significantly higher proportion of cesarean sections (85.7%) and a significantly lower proportion of spontaneous vaginal delivery (14.3%) than the sepsis and control groups. Special mode of delivery cases, including two cases of assisted vaginal delivery, one case of rivanol-induced abortion, and two cases of uterotomy, were all found in the sepsis cases. The sepsis and BSI groups harbored significantly fewer clear amniotic fluid cases than the control groups, but there was no significant difference in membrane rupture mode between the groups. The stillbirth rate of sepsis (42.2%) was higher than that of the BSI group, and no stillbirth cases were found in the control groups. Moreover, sepsis cases had the highest rates of stillbirths (20%) and artificial abortion (22%). Additionally, the newborn weights of mothers with sepsis (1,590 ± 1287.8 g) were significantly lower than those of the BSI group (2859.2 ± 966.0 g), and both were lower than those of the control group (3214.2 ± 506.4 g). The Apgar score at 1 and 5 min after delivery was applied to evaluate the physical condition of infants. The results showed that the number of normal infants in the sepsis group was significantly lower than that in the BSI and control groups. Correspondingly, the number of infants with severe asphyxia was significantly higher in the sepsis group. These results all indicated greater adverse pregnancy outcomes in sepsis patients than in BSI and other control patients. However, there was no significant difference between the sepsis-cure and sepsis-death groups.

**Table 2:** More cases of adverse fetal and maternal outcome were shown in sepsis patients than in BSI and control patients.

Characteristics	All groups					
	Sepsis (after 2010)			BSI (*n* = 31)	Control (*n* = 57)	Statistical significance
	Sepsis-death (*n* = 38 or 10^a^)	Sepsis-cure (*n* = 38)	Sepsis-total (*n* = 76 or 48^a^)			
Gestational age at delivery (weeks)	34.6 (3.4)	31.4 (7.6)	26.4 ± 9.5	32.3 ± 8.1	37.7 ± 4.0	***, †††
Mode of delivery						
Spontaneous vaginal	12 (46.2%)	18 (54.5%)	30 (50.8%)	4 (14.3%)	23 (41.8%)	**, ##, δ
Cesarean section	10 (38.5%)	14 (42.4%)	24 (40.7%)	24 (85.7%)	32 (58.2%)	***, ###, δ
Assisted vaginal	1 (3.8%)	1 (3.0%)	2 (3.4%)	0	0	ns
Rivanol-induced abortion	0	0	1 (1.7%)	0	0	ns
Uterectomy	0	0	2 (3.4%)	0	0	ns
Mode of membranes rupture						
Spontaneous	8 (57.1%)	13 (72.2%)	14 (42.4%)	6 (37.5%)	24 (43.6%)	ns
Artificial	6 (42.9%)	5 (27.8%)	19 (57.6%)	10 (62.5%)	31 (56.4%)	ns
Amniotic fluid turbidity						
Clear	5 (83.3%)	7 (63.6%)	12 (70.6%)	14 (63.6%)	52 (94.5%)	**, ††, δδδ
Grade I turbidity	1 (16.7%)	1 (9.1%)	2 (11.8%)	1 (4.5%)	0	**, †
Grade II turbidity	0	0	0	3 (13.6%)	3 (5.5%)	ns
Grade III turbidity	0	3 (27.3%)	3 (17.6%)	4 (18.2%)	0	**, ††, δδ
Pregnancy outcomes^a^						
Live births	7 (70%)	19 (55.9%)	26 (63.4%)	22 (81.5%)	55 (100%)	***, †††, δδ
Stillbirths	3 (30%)	15 (44.1%)	18 (36.6%)	5 (18.5%)	0	***, †††, δδ
Newborn weight (g)	2271.6 (726.0)	1627.8 (1306.1)	1,590 ± 1287.8	2859.2 ± 966.0	3214.2 ± 506.4	***, ###, †††
Apgar score (1 min)						
Normal (Score > 7)	8 (66.7%)	14 (43.8%)	22 (50.0%)	23 (79.3%)	55 (100%)	***, #, †††, δδ
Moderate asphyxia (4 ≤ Score ≤ 7)	0	3 (9.4%)	3 (6.8%)	1 (3.4%)	0	ns
Server asphyxia (Score < 4)	4 (33.3%)	15 (46.9%)	19 (43.2%)	5 (17.2%)	0	***, #, †††, δδ
Apgar score (5 min)						
Normal (Score > 7)	8 (66.7%)	17 (53.1%)	25 (56.8%)	24 (82.8%)	54 (98.2%)	***, #, †††, δ
Moderate asphyxia (4 ≤ Score ≤ 7)	1 (8.3%)	0	1 (2.3%)	0	0	ns
Server asphyxia (Score < 4)	3 (25.0%)	15 (46.9%)	18 (40.9%)	5 (17.2%)	1 (1.8%)	***, #, †††, δ

Data of continuous variables are presented as the mean and standard deviation (SD), and the categorical variable data are presented as counts and percentages. Symbol * represent the significance of three groups (sepsis, BSI and control). Symbols #, †, δ represent the significance of sepsis vs. BSI, sepsis vs. control, and BSI vs. control, respectively. One symbol denotes a value of *p* < 0.05. Two symbols denote a value of *p* < 0.01. Three symbols denote value of *p* < 0.001. ^a^Only sepsis-death cases enrolled after 2010 (*n* = 10) were included when calculating pregnancy outcomes to avoid deviation.

### 3.5. Organ dysfunction

Organ dysfunction, such as circulatory dysfunction, respiratory dysfunction, renal dysfunction, liver dysfunction, and disseminated intravascular coagulation (DIC), was compared between the sepsis-cure and sepsis-death groups to evaluate their death risk (Table 3 and Supplementary Table S3). The incidence of all organ dysfunctions in the sepsis-death group was higher than that in the sepsis-cure group, and circulatory dysfunction, respiratory dysfunction, and DIC showed significant differences between the groups. This confirms that organ failure is a severe situation in sepsis patients.

**Table 3:** The case number and percentage of organ dysfunction were presented and compared between the sepsis-cure and sepsis-death groups.

Characteristics	Sepsis-death (*n* = 38)	Sepsis-cure (*n* = 38)	Statistical significance
Circulatory dysfunction	21 (67.7%)	10 (32.3%)	*
Respiratory dysfunction	28 (71.8%)	11 (28.2%)	***
Renal dysfunction	8 (66.7%)	4 (33.3%)	ns
Liver dysfunction	3 (42.9%)	4 (57.1%)	ns
DIC	9 (100%)	0	**

The data are presented as counts and percentages. *Denotes *p-*value < 0.05. **Denotes value of *p* < 0.01. ***Denotes *p*-value < 0.001. Symptoms included in each group were presented in Supplementary Table S3.

### 3.6. Obstetrical critical illness

The occurrence of OCI in all patients in this study during hospitalization was summarized and analyzed. In total, 17 sepsis cases (22.4%) acquired OCI, which was similar to BSI patients (7 cases, 22.6%), and both were significantly higher than the control group (*p* < 0.01). Within the OCI of the sepsis group, 12 cases were postpartum hemorrhage/hemorrhagic shock, which was significantly higher than that in BSI (2 cases). The occurrence of fetal distress was significantly higher in the BSI group than in the sepsis group (5 cases vs. 2 cases). In addition, two cases of acute fatty liver during pregnancy and one case of amniotic fluid embolism was found in the sepsis group, while none were found in the BSI group. As expected, no OCI occurred in the control group. In addition, significantly more OCI cases presented in the sepsis-death group than in the sepsis-cure group (13 cases vs. 3 cases). Additionally, 10 out of 13 cases of postpartum hemorrhage/hemorrhagic shock were discovered in the sepsis-death group, which was significantly higher than that in the sepsis-cure group. Hence, OCI, especially postpartum hemorrhage/hemorrhagic shock, could be a high-risk factor.

## 4. Discussion

In septic pregnant women, their mean arterial blood pressure remains significantly reduced, leading to fetal hypoxia. Furthermore, under stress, increased secretion of maternal catecholamines causes an increase in uterine vascular resistance, and blood is redistributed to vital maternal organs, which further compromises fetal well-being [Chau, 2014 (17)]. Sepsis is one of the major diseases that require patients in the pregnancy and postpartum periods to be sent to the ICU, leading to high maternal and perinatal mortality or maternal near miss (18, 19). Pregnancies complicated by antepartum sepsis were associated with higher odds of placental dysfunction (20). Pregnancy loss is a frequent outcome of maternal sepsis. Knowles (12) reported the fetal outcome following maternal sepsis in the three trimesters, and the live-born and discharged home infants were 88.1% (238/270 cases). Fetal death occurs mainly in middle pregnancy and is mainly associated with chorioamnionitis. The fetal infection rate was 81.8% (18/22 cases) (12). In the current study, stillbirth accounted for 20% (9 cases), and artificial abortion occurred in 10 cases (22%).

The fetal oxygenation, metabolic exchange, and fluid status are dependent on the maternal circulation. In patients with BSI, metabolisms of pathogens in the bloodstream damage the endothelium of blood vessels, increase capillary permeability, cause tissue edema, and reduce the effective circulating blood volume of the mother. Additionally, the immune system produces inflammatory cytokines, nitric oxide, and other mediators that cause vasodilation and lower blood pressure. The reduction in circulating blood volume and blood pressure leads to a decrease in uterine-placental blood flow, resulting in fetal hypoxia (21, 22). Considering the pathological similarities and differential disease severity between bloodstream infection (BSI) and sepsis, we have included BSI pregnant women as controls, in addition to healthy pregnant women, to better reflect the pre-onset state of sepsis.

The onset period was compared among sepsis, BSI, and control patients. Both BSI and sepsis occurred during the postpartum period, but no control cases presented in postpartum period, which indicated that the birth wound may be the main cause of infection. Except for the postpartum period, sepsis also occurred throughout every stage of pregnancy, inferring that the incidence of sepsis was affected by various factors other than wound infection, which is more complex than that of BSI.

Bloodstream infection can occur secondary to the infection of specific body sites or be considered primary infection when no specific infection sites are found (23). Either of them is an important process leading to sepsis, and the infection site is a crucial factor driving the decision regarding the treatment strategy (24). Hence, the infection sources were analyzed in the current study. The results showed that the number of GTIs in BSI pregnancies was significantly higher than that in sepsis pregnancies, which may be due to the extensive experience of obstetricians in the treatment of intrauterine infection. BSI can be effectively cured with appropriate treatment, which will further avoid the development of sepsis. In contrast, pneumonia was more likely to occur in sepsis cases and sepsis-death groups. This may remind obstetricians to pay more attention to the non-reproductive tract infection circumstance.

Pregnancy has long been considered an immunocompromised state and has recently been recognized as a state of immunomodulation. A competent immune response is crucial to protect the mother and fetus (25, 26). Microbial infection is the most common cause of septic shock which produces endotoxins and exotoxins that generate a heightened inflammatory response, leading to increased endothelial dysfunction and vascular permeability (27, 28). The virulence of microorganisms could determine the severity of the manifestation and the treatment strategies and outcomes. In the current study, microorganisms were cultured and identified from the blood, urine, skin, etc. We found that *E. coli* was the most frequent bacterium, which is consistent with previous studies. Vasco et al. reported that *E. coli* was the most common pathogen of sepsis in the pregnancy and postpartum periods in 2019 (4). Knowles et al. found that *E. coli* (103/276 strains, 37.3%) was the most common pathogen of sepsis in the pregnancy and postpartum periods, followed by Group B streptococci (57/276 strains, 20.7%) (12). Abir et al. reported that of 35 sepsis cases in pregnancy, bacterial culture was negative in 50% of the cases. *E. coli* was detected in 27% of the cases, and *Staphylococcus aureus* was detected in 9% of the cases (29). In addition, *Candida* is the most common fungus cultured from sepsis patients and has long been recognized in recent years. Although gram-negative bacteria infectious cases have been reported as the largest group, lower proportion of gram-negative bacteria infectious cases in sepsis and even lower in sepsis-death group was shown in this study than previous studies (28, 30). Moreover, a higher proportion of gram-positive bacteria was present in the sepsis-death group than in the sepsis-cure group. Similarly, Duan et al. reported that greater adverse maternal and fetal outcomes, such as premature delivery rate, miscarriage rate, and fetal/neonatal mortality rate, were all higher in gram-positive bacterial infection cases. They all inferred that more attention should be given to gram-positive bacterial infection cases due to their higher threat.

Organ dysfunction is one of the criteria for the diagnosis of sepsis, which requires a sequential organ failure assessment (SOFA) score ≥ 2 with a suspicion of infection. Organ dysfunction mainly includes circulatory dysfunction, respiratory dysfunction, renal dysfunction, liver dysfunction, and DIC. In the current study, the proportions of circulatory dysfunction, respiratory dysfunction, and DIC were significantly higher in the sepsis-death group than in the sepsis-cure group. Respiratory infection or pneumonia, which may result in respiratory dysfunction, was proven to be the most common symptom in sepsis death cases in a previous study (31) and this study. Hence, early diagnosis and appropriate treatment of respiratory infection or pneumonia would reduce the likelihood of progression to sepsis and death. In addition, DIC is associated with death from sepsis (32). Therefore, extra care should be taken for severe postpartum hemorrhage, which may lead to DIC, to avoid adverse outcomes.

Obstetric critical illness can be caused by complex factors and various comorbidities, and its incidence in the United States is approximately 0.47–0.75% (33). Sepsis may also result from or result in OCI, such as severe obstetric hemorrhage, amniotic fluid embolism, acute fatty liver of pregnancy, congestive heart failure, cardiac arrest, and severe trauma (2). In this study, 24 cases of OCI were complicated with infection. Severe blood loss stimulates excess inflammatory cytokine production and leads to systemic inflammatory response syndrome (SIRS), multiple organ dysfunction, and progression to sepsis (34). The probability of postpartum hemorrhage/hemorrhagic shock complicated with sepsis (85.71%) was significantly higher than that of the BSI group (14.29%), which was consistent with the results of previous studies that indicated an increased risk of sepsis due to postpartum hemorrhage (OR, 1.17; 95% CI, 1.08–1.28) (35). In addition, fetal distress will occur due to uterine infection after maternal bloodstream infection due to bacterial transformation through blood circulation. Our results showed that fetal distress was more likely to cause BSI than sepsis (5 cases vs. 2 cases). ACOG suggests that termination of pregnancy is a better choice for fetal distress that is unable to improve [Obstetricians and Gynecologists, 2021 (14)]. Proper pregnancy termination may contribute to anti-infectious and supportive treatment outcomes and further prevent the occurrence of sepsis.

The present study has limitations to consider, such as population characteristics that were not always well described. The cross-sectional design of our study precluded the establishment of a causal relationship between the identified risk factors and sepsis. All patients could not be included so that it was not possible to have maternal mortality and the true incidence for each sepsis-related condition. Bias could also be introduced by the absence of the cured sepsis cases before 2010. The size of our study population was relatively small, resulting in a lack of significant differences between the sepsis-cure and sepsis-death groups across many characteristics.

## 5. Conclusion

This study was a retrospective study on sepsis cases to explain the related risk factors by comparing them with BSI and control maternities. Significantly more sepsis and BSI cases occurred during the postpartum period, suggesting that a higher infection risk may be caused by birth wounds. GTI and pneumonia were the two most common infection sources, while higher proportions of pneumonia cases presented in sepsis and sepsis-death when compared with BSI and sepsis-cure, respectively, indicating its higher risk and serving as a reminder that obstetricians should care more about the nonreproductive-tract-infection-circumstance of sepsis patients. More gram-positive bacteria and fungi were cultured from the sepsis group than from the BSI group, as well as from the sepsis-death group than from the sepsis-cure group, which suggests that more attention should be given to the treatment of gram-positive bacteria and fungi. As expected, greater adverse maternal and fetal outcomes, such as higher mortality, earlier gestational age at delivery, and lower newborn weight, were observed in the sepsis group. This study offered some potential pathogenesis and mortality risk factors for sepsis, which may inspire the treatment of sepsis in the future.

## Data availability statement

The original contributions presented in the study are included in the article/Supplementary material, further inquiries can be directed to the corresponding authors.

## Ethics statement

This article does not contain potentially identifiable images or data.

## Author contributions

PL and SF conceived and designed the research. YL, NW, ZX, TL, RZ, and WZ carried out the research and collected the samples. PL and XW managed the database, rechecked the data, and helped with the original draft preparation. XZ analyzed the data and drafted the manuscript. SF reviewed and revised the manuscript and had primary responsibility for the final content. All authors have read and agreed to the published version of the manuscript.

## Funding

This research was supported by the National Natural Science Foundation of China (82171676 and 82201793), Science and Technology Planning Project of Shenzhen Municipality (JCYJ20220530160206014), and the Scientific Research Foundation of Peking University Shenzhen Hospital (KYQD202100X and KYQD2022111).

## Conflict of interest

The authors declare that the research was conducted in the absence of any commercial or financial relationships that could be construed as a potential conflict of interest.

## Publisher’s note

All claims expressed in this article are solely those of the authors and do not necessarily represent those of their affiliated organizations, or those of the publisher, the editors and the reviewers. Any product that may be evaluated in this article, or claim that may be made by its manufacturer, is not guaranteed or endorsed by the publisher.
